# Longitudinal evaluation of visual function and structure for detection of subclinical Ethambutol-induced optic neuropathy

**DOI:** 10.1371/journal.pone.0215297

**Published:** 2019-04-17

**Authors:** Ki Won Jin, Joo Yeon Lee, Soolienah Rhiu, Dong Gyu Choi

**Affiliations:** Department of Ophthalmology, Hallym University College of Medicine, Seoul, Republic of Korea; Roskamp Institute, UNITED STATES

## Abstract

**Purpose:**

To longitudinally evaluate the visual function and structure of patients taking ethambutol by various modalities and identify useful tests for detection of subclinical ethambutol-induced optic toxicity.

**Methods:**

This retrospective study enrolled 84 patients with newly diagnosed tuberculosis treated with ethambutol. Best-corrected visual acuity (BCVA), color vision, contrast sensitivity, fundus and retinal nerve fiber layer (RNFL) photography, automated visual field (VF) test, and optical coherence tomography (OCT) were performed: prior to starting; every month during administration, and 1 month after stoppage. We longitudinally compared visual function and structure with the baseline and identified the occurrence of subclinical toxicity.

**Results:**

BCVA, color vision, and contrast sensitivity showed no change from the baseline. Mean temporal RNFL thickness was significantly increased at 6 months (p = 0.014). Subclinical toxicity was found in 22 eyes of 14 patients (i.e., 13% of 168 eyes), in the forms of VFI decrease (VF index, 9 eyes of 6 patients), quadrant RNFL thickness increase (5 eyes of 4 patients), and VF pattern defect (12 eyes of 6 patients). 73% of the patients showed recovery to the baseline at 1 month post-stoppage. The risk factors for occurrence of subclinical toxicity were age, cumulative dose, and medication duration.

**Conclusion:**

Mean temporal RNFL thickness increased after administration. The VFI, quadrant RNFL thickness, and VF pattern defect could prove useful in assessment of subclinical toxicity. Medication duration was shown to be a strong risk factor for occurrence of subclinical toxicity.

## Introduction

Ethambutol, first introduced in 1961 as a bacteriostatic agent for *Mycobacterium tuberculosis*, remains the primary therapy for infections caused by Mycobacterium tuberculosis and avium complex. Since Carr and Henkind’s inaugural 1962 report of ethambutol-induced optic neuropathy [1], ethambutol has become a well-recognized cause of toxic optic neuropathy, with dose-related severity [2, 3]. The exact mechanism of Ethambutol ocular toxicity remains to be established; however, it has been known that it might result from decreased levels of copper in mitochondria or from accumulation of zinc in lysosomes of retinal ganglion cells [4, 5]. Ethambutol-induced optic neuropathy incidence has been reported to be above 1% [6, 7]. The risk is below 1% at doses less than 15mg/kg/day, and is reported to be increased with higher doses of 20 and 25mg/kg/day to 3 and 5–6%, respectively [8]. Previous studies have recommended maintenance of the ethambutol dose as close to 15mg/kg/day as possible [9] and, for patients administered larger daily dosages, obtainment of baseline visual examination values and performance of monthly examinations [10].

Although ethambutol-induced optic neuropathy is known to be reversible [10], complete recovery is not always possible, in which cases permanent visual impairment is incurred [11–12]. The degree of reversibility accords with the time of detection; in fact, early detection and immediate termination of therapy are the only effective means of preventing progression and facilitating recovery [3, 13]. Screening methods for subclinical optic toxicity have not yet been established [9]; most previous studies, meanwhile, have focused on the review of clinical optic neuropathies occurring after signs and symptoms of toxicity have become manifest [14–22].

The specific purpose of the present study was to longitudinally evaluate visual function and structure using various modalities in order to determine the most useful testing methods for detection of subclinical ethambutol-induced optic neuropathy.

## Materials and methods

### Study design

This retrospective study enrolled 114 consecutive patients with newly and definitely diagnosed pulmonary and extra-pulmonary tuberculosis between March 2014 and March 2016 at three hospitals affiliated with Hallym University. The patients had been diagnosed in the Pulmonology or Infection Clinics within the Internal Medicine department and referred to Neuro-ophthalmology Clinics prior to starting anti-tubercular treatment including ethambutol. As a standard of care for all patients receiving ethambutol, ophthalmic examinations were performed while taking the medicine according to the schedules.

All of the patients were selected in accordance with the following exclusion criteria: history of optic neuropathy or retinal disease, history of intraocular or refractive surgery other than cataract extraction, media opacity other than incipient cataract, best-corrected visual acuity (BCVA) < 20/50, intraocular pressure (IOP) > 21mmHg, pre-existing visual field defects, and history of taking any non-ethambutol drugs known to cause ocular toxicity. This study was approved by the Institutional Review Board (IRB) of Hallym hospitals and adhered to the tenets of the Declaration of Helsinki. Given the retrospective study design with medical chart, IRB approved the current study to be exempted from obtaining written consent. We had verbal informed consent from the participants and included only data of the participants with consent. All of the data were newly collected for the current study and have never been published previously.

### Patient evaluation

We performed a detailed ophthalmology history taking and reviewed the Internal Medicine department’s medical records for each participant. The first examination was performed as a baseline test (prior to the start of drug administration) when the patients first visited our Neuro-ophthalmology Clinic. After initiation of ethambutol administration, the patients visited the clinic each month while taking the drug, and then again 1 month after stoppage. At each visit, ophthalmic examinations were performed, including slit-lamp examination, fundoscopy, BCVA, color-vision testing by Ishihara color plates, contrast sensitivity testing by the Mars Letter Contrast Sensitivity chart (Mars Perceptrix, Chappaqua, NY, USA), fundus photography, and retinal nerve fiber layer (RNFL) photography. We assessed the patterns of visual field (VF) defect and abnormalities in VF global indices including mean deviation (MD), pattern standard deviation (PSD), and VF Index (VFI) using automated perimetry by Humphrey Field Analyzer (Central 24–2 pattern; Carl Zeiss Meditec, Dublin, CA, USA). Any test result of low reliability (false positive or false negative > 15%, or fixation loss > 20%) was excluded from the VF test analysis. We carefully differentiated true defects from temporary variations by comparison of all available sequential VF tests in those with VF pattern changes. Peripapillary RNFL thickness was measured for the 4 quadrants and the global (360°) average using Cirrus high-definition spectral-domain optical coherence tomography (HD SD-OCT; Carl Zeiss Meditec, Dublin, CA, USA). Only OCT test results with a signal strength above 7 and good centration were included in the subsequent analysis. We removed all data for cases in which structural evaluation of the automatic segmentation of the RNFL was incorrect.

### Indentifying subclinical toxicity

Subclinical change or toxicity was defined as the lack of recognizable clinical symptoms or signs, but with any significant changes on ophthalmic examinations including color-vision, contrast sensitivity testing, fundus photography, RNFL photography, VF testing, or OCT. Subclinically significant changes in numeric characteristics such as color vision, contrast sensitivity, VF global indices, and RNFL thickness by OCT were defined as those showing a value difference (increase or decrease) greater than 2 SD from the baseline, as in a previous report by Menon et al [23]. Clinical assessment of non-numeric parameters such as fundus photography, RNFL photography and VF defects was performed by two independent observers (K.W.J., D.G.C.) masked to the other test results. The results were confirmed and considered subclinically significant only if those two observers concurred.

### Statistical analysis

All of the analyses were performed using statistical software (SPSS for Windows V.12.0.K, Chicago, IL, USA). The sampling distribution of the study, which reflects a large sample size (> 30), approximates a normal distribution according to the Central Limit Theorem. The test results at each visit were compared with the baseline via paired-t test. The changes of parameters along the time course were examined via repeated measures ANOVA. Multivariate logistic regression analysis was employed to determine the associations of the demographic and clinical features with occurrences of subclinical change. Cox proportional hazard model was performed to deduce the hazard ratio of risk factors at a particular time. A p value less than 0.05 was considered statistically significant.

## Results

Among the 114 patients initially enrolled, a total 168 eyes of 84 patients were included in the present study. Fifteen patients were found to have taken ethambutol before the initial visit due to delayed referral, and 13 patients were lost to follow up after the initial examination. Two patients were excluded because of poor cooperation. The OCT result of 1 patient with high myopia was excluded from analysis because of misalignment, and the color-vision tests of 3 patients with red-green color blindness were discarded.

The baseline characteristics of the participants are provided in Table 1. The mean age was 45.50 ± 17.17 years (range: 16–84). The mean daily dose of ethambutol was 14.72 ± 3.07 mg/day/kg (range: 6.35–23.08) over a mean administration duration of 4.31 ± 2.42 months (range: 1–9). The other drugs included in the treatment regimen were isoniazid, rifampicin, and pyrazinamide. Most of the participants had pulmonary tuberculosis (66 patients, 79%), followed by pleural effusion (8 patients, 10%) and lymphadenitis (7 patients, 8%). The other involved organs were the kidneys (1 patient), the GI tract (1 patient), and the meninges (1 patient). Five patients (6%) showed a decreased (i.e., <60 mL/min/1.73m^2^) estimated glomerular filtration rate (GFR). The daily dose/weight of 5 patients with reduced GFR was 13.72 ± 0.85 (range: 12.53–14.60), which showed no significant difference with normal GFR subjects (p = 0.332, Mann-Whitney U test). Among the total cohort of 84 patients, 21 were followed 1 month after drug stoppage. The patients’ baseline visual functions and structures are provided in Table 2.

**Table 1:** Demographic and clinical characteristics of participants.

**Age (years)**	45.50 ± 17.17 (range: 16–84)
**Sex (male/female)**	39 (47.1%) / 45 (52.9%)
**Follow-up duration (months)**	4.69 ± 2.24 (range: 2–10)
**Ethambutol administration**	
** Daily dose/weight (mg/day/kg)**	14.72 ± 3.07 (range: 6.35–23.08)
** Cumulative dose (mg)**	3531.25 ± 2018.11 (range: 600–8800)
** Medication duration (months)**	4.31 ± 2.42 (range: 1–9)
**Other medication dose**	
** Isoniazid (mg)**	303.27 ± 49.82 (range: 150–400)
** Rifampicin (mg)**	546.43 ± 72.09 (range: 450–600)
** Pyrazinamide (mg)**	1406.25 ± 249.95 (range: 150–2000)
**Involved organ**	
** Lung**	66 (79%)
** TB pleural effusion**	8 (10%)
** TB lymphadenitis**	7 (8%)
** Others**	3 (4%)
**Underlying disease**	
** DM**	10 (12%)
** HTN**	8 (10%)
** Cancer**	1 (1%)
** Decreased GFR (mL/min)**	5 (6%)

TB: Tuberculosis, DM: Diabetes mellitus, HTN: Hypertension, GFR: estimated Glomerular filtration rate

**Table 2:** Baseline visual function and structure.

Parameters	Average ± SD	Ranges
**Visual acuity (logMAR)**	0.03 ± 0.07 (n = 166)	0.00–0.40
**Color test (/24)**	22.42 ± 4.37 (n = 166)	2–24
**Contrast sensitivity**	1.57 ± 0.17(n = 166)	0.72–1.84
**VF**	(n = 158)	
** MD (dB)**	-2.18 ± 2.56	-14.04–2.50
** PSD (dB)**	2.47 ± 1.58	0.94–12.31
** VFI (%)**	96.69 ± 4.84	69–100
**RNFL thickness (μm)**	(n = 168)	
** Average**	101.53 ± 13.89	73–144
** Superior**	126.56 ± 16.85	76–179
** Inferior**	129.55 ± 21.59	92–176
** Temporal**	78.81 ± 16.55	53–140
** Nasal**	73.02 ± 15.34	49–140

n = number. VF: Visual field, MD: Mean deviation, PSD: Pattern standard deviation, VFI: Visual field index, RNFL: Retinal nerve fiber layer

None of the patients complained of blurred vision or any other subjective ocular symptoms at any time during the study, and so no clinical ethambutol-induced optic neuropathies occurred during the follow-up visits. By repeated measures ANOVA, none of the parameters showed significant difference along the time course (p>0.05). BCVA, color vision, and contrast sensitivity showed no significant change from the baseline throughout the study period (p>0.05).

The global indices of VF were improved from the baseline at every visit, with statistical significance at some points, possibly due to the learning effect, as shown in Table 3 (p<0.05). In mean temporal RNFL thickness, a significant change was observed at 6 months (p = 0.014) (Table 4). There was no significant change in mean RNFL thickness in the superior, inferior or nasal quadrants. Likewise, the 360°-average RNFL thickness showed no significant change relative to the baseline. Sub-analyses of VF indices and RNFL performed with the data of patients who had visited at a particular time are provided in S1 and S2 Tables, respectively.

**Table 3:** Serial measurement of visual field global indices by Humphrey Field Analyzer.

	Baseline (n = 158)	1 month (n = 139)	2 months (n = 116)	3 months (n = 82)	4 months (n = 70)	5 months (n = 60)	6 months (n = 40)	7 months (n = 16)	8 months (n = 14)	After stoppage (n = 34)
**MD (dB)**										
**Mean**	-2.18	-1.87	-1.96	-1.41	-1.45	-1.89	-1.02	-1.52	-1.75	-1.12
**SD**	2.56	2.44	2.34	2.40	2.30	2.24	2.05	2.34	3.16	1.69
p value*		0.664	**0.011**	**0.000**	**0.000**	**0.001**	**0.000**	**0.005**	**0.010**	**0.000**
**PSD (dB)**										
**Mean**	2.47	2.43	2.51	2.28	2.07	2.02	1.70	2.04	1.85	2.02
**SD**	1.58	2.01	1.73	1.39	1.08	0.90	0.60	0.92	0.61	1.18
p value*		0.327	0.603	**0.009**	**0.001**	**0.005**	**0.023**	0.219	0.226	**0.000**
**VFI (%)**										
**Mean**	96.69	96.83	96.73	97.09	97.53	97.18	98.28	97.75	97.07	98.38
**SD**	4.84	4.37	4.29	4.07	3.34	3.99	3.06	3.61	5.97	2.05
p value*		0.441	0.335	**0.003**	**0.004**	**0.029**	**0.017**	0.081	0.112	**0.005**

MD: Mean deviation, PSD: Pattern standard deviation, VFI: Visual field index. n = number

*: Paired-T test

**Table 4:** Serial measurement of retinal nerve fiber layer thickness by Cirrus high-definition spectral-domain optical coherence tomography.

	Baseline (n = 168)	1 month (n = 152)	2 months (n = 124)	3 months (n = 88)	4 months (n = 76)	5 months (n = 60)	6 months (n = 40)	7 months (n = 16)	8 months (n = 14)	After stoppage (n = 42)
**Average**										
**Mean (μm)**	101.53	101.93	101.51	99.89	100.11	99.02	105.65	96.19	97.00	97.80
**SD**	13.89	12.63	13.43	13.41	13.19	11.02	15.38	11.49	11.37	10.86
p value*		0.313	0.708	0.429	0.502	0.733	0.683	0.464	0.303	0.569
**Superior**										
**Mean (μm)**	126.56	127.01	126.14	123.54	122.87	122.96	131.34	118.56	120.17	119.63
**SD**	16.85	19.95	19.20	20.12	19.45	16.81	20.38	16.93	10.87	17.13
p value*		0.330	0.572	0.863	0.152	0.603	0.923	0.157	0.139	0.827
**Inferior**										
**Mean (μm)**	129.55	129.38	130.01	126.38	127.34	123.54	135.53	126.75	127.83	123.18
**SD**	21.59	22.91	22.00	21.95	20.97	19.59	23.12	19.85	15.71	20.22
p value*		0.694	0.172	0.804	0.581	0.705	0.291	0.544	0.110	0.319
**Temporal**										
**Mean (μm)**	78.81	78.41	78.61	80.32	78.34	78.71	82.76	75.00	71.17	78.68
**SD**	16.55	16.62	16.06	18.68	20.97	17.22	19.43	15.54	16.95	15.22
p value*		0.530	0.538	0.060	0.794	0.102	**0.014**	0.840	0.699	0.116
**Nasal**										
**Mean (μm)**	73.02	72.72	71.92	70.02	71.97	70.75	74.29	67.19	70.00	69.28
**SD**	15.34	15.50	13.71	14.20	13.50	15.04	16.69	16.99	16.25	17.12
p value*		0.812	0.933	0.428	0.354	0.274	0.975	0.540	0.557	0.640

n = number

*: Paired-T test

According to the definition of subclinical toxicity, 0.14 in visual acuity, 8 in color-vision, and 0.32 in contrast sensitivity corresponded to 2 SD differences from the average of the baseline. Visual acuity, color vision and contrast sensitivity showed no subclinically significant change at any point of the study. As for the VF indices, 5.12dB in MD, 3.16 dB in PSD, and 9.68% in VFI corresponded to 2 SD differences from the mean of the baseline. None of subjects at any point showed subclinically significant changes in MD or PSD. However, VFI showed subclinically significant decrease in 9 eyes of 6 patients. Although bilateral changes of VFI were observed in all 6 patients, the contralateral eyes of 3 patients did not reach the subclinically significance threshold of 2 SD difference. The thickness changes of 27um in average, 33um in superior, 43um in inferior, 33um in temporal, and 30um in nasal quadrant corresponded to 2 SD differences from the mean of the baseline. A subclinically significant increases in 5 eyes of 4 patients were observed, and none of the subject showed decrease. The affected quadrants were the superior in 3 eyes, the temporal in 1 eye, and the nasal in 1 eye. Bilateral superior changes were observed in 1 patient. The fundus and RNFL photographies were not significantly different from the baseline exam. In regards to VF, There were VF defects in 12 eyes (bilateral eyes of 6 patients), in the forms of peripheral constriction (4 eyes) and superior or inferior altitudinal defect (8 eyes). All of the patients showed bilateral and symmetric defects.

Subclinical ethambutol-induced optic neuropathy was found in a total of 22 eyes of 14 patients (i.e., 13% of 168 eyes). Four eyes of 3 patients showed both VFI and VF pattern changes. Among 11 eyes of 7 patients followed at 1 month after stoppage, recovery to the baseline level was observed in 8 eyes (73%). Seven eyes of 5 patients with VFI decrease were followed at 1 month after drug stoppage, and the values in all observed cases returned to the baseline levels. Only 1 patient with unilateral temporal quadrant RNFL change visited 1 month after stoppage, showing persistent change, and 2 subjects (4 eyes) with altitudinal defect visited at 1 month post-stoppage, and all showed persistent field defects.

The risk factors associated with occurrence of subclinical ethambutol-induced optic neuropathy were older age (, 95% CI 1.013–1.145, p = 0.018), lower cumulative dose (OR 0.996, 95% CI 0.993–0.998, p = 0.002), and longer medication duration (OR 19.384, 95% CI 2.768–135.747, p = 0.003) (Table 5).

**Table 5:** Factors associated with occurrence of subclinical ethambutol-induced optic neuropathy.

	Odds ratio	95% Confidence interval	P value*
**Age (years)**	1.077	(1.013, 1.145)	**0.018**
**Sex**	2.327	(0.521, 10.402)	0.269
**Ethambutol administration**			
** Daily dose/weight (mg/day/kg)**	1.229	(0.853, 1.770)	0.269
** Cumulative dose (mg)**	0.996	(0.993, 0.998)	**0.002**
** Medication duration (months)**	19.384	(2.768, 135.747)	**0.003**
**Involved organ**	NA	NA	0.407
**Underlying disease**			
** DM**	0.407	(0.049, 3.392)	0.406
** HTN**	14.282	(0.319, 639.090)	0.170
** Cancer**	108312330754.841	(0.000, NA)	0.999
** Decreased GFR (mL/min)**	0.419	(0.012, 14.969)	0.633

DM: Diabetes mellitus, HTN: Hypertension, GFR: estimated Glomerular filtration rate

*: Multivariate logistic regression analysis

## Discussion

Ethambutol-induced optic neuropathy is typically described as bilateral, progressive, and painless visual loss that might accompany early diminishment of color vision [10, 22, 24]. None of the present study’s patients showed any decrease in BCVA, color vision or contrast sensitivity during the study period. This finding does not coincide with Salmon et al., who reported diminished contrast sensitivity in patients receiving ethambutol treatment (38.2% at 3 months, 36.7% at 6 months) [25]. However, our result was consistent with Menon et al. and Kim and Park, who likewise reported “no changes” in visual acuity, color vision or contrast sensitivity [23, 26]. Whereas we could not find decreases in BCVA, color vision or contrast sensitivity, though subclinical changes could be found in VF and OCT. We found the subclinical toxicities in the forms of VFI decrease (9 eyes of 6 patients, 5%), quadrant RNFL thickness increase (5 eyes of 4 patients, 3%), and VF pattern defect (12 eyes of 6 patients, 7%) in the current study, so these tests could prove useful in assessment of subclinical toxicity. In other words, it could be said that VF and OCT are more sensitive for detection of subtle changes, and that on the contrary, BCVA, color vision and contrast sensitivity can be thought to decrease when clinical toxicity is imminent. It is recommended that all patients with Ethambutol administration be screened monthly with OCT and VF if such tests are available. As we performed clinic-based monthly examinations, we used Ishihara color plates, which are pseudo-isochromatic plates designed for screening, along with the Mars Letter Contrast Sensitivity chart (Mars Perceptrix, Chappaqua, NY, USA), a simple and convenient letter test. A more sensitive and time-consuming test, such as the 100-hue test for color or a computer-based test for contrast sensitivity, could be adopted in a future prospective study. Visual evoked potential (VEP) can also be useful in the detection of subclinical toxicity. Delay of mean latency of the P100 wave after Ethambutol administration has been noted in previous studies, whereas there is debate on changes in P100 amplitude [23, 26–28]. The amplitude was not significantly changed in the studies of Menon et al. and Kim and Park; however Yiannikas et al. reported both latency and amplitude changes [23, 26, 28]. In the present study, temporal RNFL thickness showed post-administration increases at 6 months. Similarly, Kim and Park’s prospective study revealed significant post-administration increases in temporal and inferior RNFL thickness at 5 months [26]. After Zoumalan et al. first reported RNFL thickness change in ethambutol-induced optic neuropathy, several retrospective OCT-based studies on RNFL thickness have similarly reported decreases in temporal RNFL thickness over the course of long-term follow ups compared with the baseline or healthy controls after ethambutol-induced optic neuropathy [14–16, 19]. The results of prospective studies, however, have been various. Menon et al. and Gümüş and Öner reported decreases in RNFL, whereas Han et al. reported no significant changes, and Kim and Park, as noted above, showed increases similar to our present results [23, 26, 29, 30]. One possible explanation of increase is that ethambutol causes mitochondrial disturbance, which results in decreased levels of energy for axonal transport. Such damage is particularly serious in the papillomacular bundle’s small-caliber axons (parvo-cellular RGC axons), and in the early stage, mild swelling of the papillomacular bundle can be observed, as in a report based on an in vitro Leber’s hereditary optic neuropathy (LHON) -mimicking mice model [31–33]. Although evaluation of mean quadrant RNFL thickness can be useful to the observation of change trends, the results cannot be applied to individual clinical situations. Thus, clinicians should monitor carefully for significant peripapillary RNFL change (i.e., > 2 SD). According to either our present data (27um in average, 33um in superior, 43um in inferior, 33um in temporal, and 30um in nasal quadrant) and those of the previous report (20 um in average), the extent of 20–30 μm increases or decreases corresponded to a 2 SD difference from the mean of thickness at the baseline, and should be regarded as significant changes. RNFL increase needs to be monitored with special care, as it is expressed in white color in the peripapillary RNFL report [23, 26].

The most commonly reported visual field defect in cases of ethambutol-induced toxicity is central or ceco-central scotoma [24]; meanwhile, less commonly, peripheral constriction [22, 23], altitudinal defect [34], and bitemporal hemianopsia have been reported [20]. In the current study, 12 eyes showed VF-pattern defects in the forms of bilateral symmetric peripheral constriction and altitudinal defect.

Recent studies on detection of ethambutol-induced optic neuropathy have focused on objective and quantitative measure of OCT; however, VF testing can provide useful information considering the fact that it is not clear whether structural change occurs in advance of functional defect. In our study, all of the VF indices improved from the baseline, due possibly to the learning effect. Clinicians should focus more on subtle changes in VF patterns than on global VF indices, and when in doubt about the VF pattern, they should repeat the test.

In the current study, subclinical ethambutol-induced optic neuropathy was found in a total of 22 eyes of 14 patients (i.e., 13% of 168 eyes), 73% of which eyes recovered to the baseline level. VF defects remained at 1 month after stoppage in all of the observed cases. The study considered that subclinical changes are not uncommon among patients with Ethambutol administration, and that unrecovered cases could exist. We did not stop administration of Ethambutol in the subclinical cases, and none of these patients developed clinical Ethambutol-induced optic neuropathy until 1 month after administration. Thus, the results of the present study suggest that close follow-up on a monthly basis is required for patients with subclinical toxicities, though cessation of administration is not recommended. Nonetheless, we need to elucidate the implications of subclinical toxicity for actual occurrence of clinical toxicity in prospective studies with large numbers of patients in the future

Risk factors for ethambutol-induced optic toxicity have been thoroughly studied. Age, renal dysfunction, dosage of ethambutol [6, 9], and hypertension are known to be positively correlated with risk of toxicity [35]. The factors associated with subclinical toxicity in the present study were older age (OR 1.077, 95% CI 1.013–1.145, p = 0.018), lower cumulative dose (OR 0.996, 95% CI 0.993–0.998, p = 0.002), and longer medication duration (OR 19.384, 95% CI 2.768–135.747, p = 0.003). However, the odds ratio for age and cumulative dose was near 1, indicating that these exposures were not strongly associated with occurrence. Medication duration, contrastingly, showed a strong odds ratio, based on which, we speculated as to whether long-term drug exposure is strongly associated with the occurrence of subclinical toxicity. We believe that there should be thorough monthly examinations in the patients who were expected to have long-term administration. Decreased GFR was not a risk factor in the current study. We infer that all 5 patients had decreased GFR (i.e., <60 mL/min/1.73m2), but with no severe reduction below 30 mL/min/1.73m2, and that no one received a renal-adjusted dose. We further performed the Cox proportional hazard model to deduce the hazard ratio at a particular time, by allotting the elapsed time before a subject was identified as a case of subclinical toxicity for a time variable and each time period for an end point. As a result, the occurrence of subclinical toxicity was affected by longer medication duration and lower cumulative dose until 3 months, but not after, including 1 month after stoppage (medication duration HR 6.823, 95% CI 1.939–24.004, p = 0.003 at 1 month; HR 7.391, 95% CI 2.499–21.857, p = 0.000 at 2 months; HR 4.235, 95% CI 1.254–14.307, p = 0.020 at 3 months, cumulative dose HR 0.997 95% CI 0.999–0.995, p = 0.004 at 1 month; HR 0.997 95% CI 0.999–0.995, p = 0.000 at 2 months; HR 0.998 95% CI 1.000–0.996, p = 0.020 at 3 months). We speculated that the low attendance rate of the subjects at the latter part of the follow-up affected the relative risk, which could not be controlled in this retrospective study. A future prospective study could elucidate the effect of the risk factors according to time course.

In this study, the incidence of clinical toxicity could not be rated, because no patient complained of clinical symptoms. However, we could assume that the incidence would be lower than 1.2% (1/84), which is compatible with the results of previous studies [6, 7].

Unlike most of the previous studies, which investigated small sample sizes for short periods of time, the present study involved a large number of samples and reviewed subjects for long-duration longitudinally according to a retrospective design. The longer follow up was required, because ocular toxicity does not usually occur during the first 2 months, but generally is present between 4 and 12 months post-administration [24]. Notwithstanding our study’s retrospective design, it has some of the positive aspects of a prospective study, as we routinely performed baseline and monthly follow-up examinations for all newly diagnosed tuberculosis patients from an Internal Medicine department.

This study has some limitations. None of the participants experienced clinical symptoms, and therefore, incidence of clinical optic neuropathy after subclinical change could not be ruled out. We did not stop administration of ethambutol in the subclinical cases, and none of these patients developed clinical ethambutol-induced optic neuropathy until 1 month after administration. Thus, the implications of subclinical ethambutol-induced toxicity for actual occurrence of clinical toxicity remain to be elucidated in another long-term, prospective studies. Secondly, we could not evaluate the patients for a sufficient span of time after stoppage of drug administration. Given the retrospective study design, we were not able to control the follow-up visitation, and so half of subjects with subclinical changes failed to visit after stoppage of administration (7 of 14 patients), and we were also were unable to collect data beyond 1 month after stoppage. In reversible cases, the resolution of ethambutol-induced optic toxicity typically occurs 3 months after cessation [36]. With a longer follow-up period, subclinical changes in VF pattern and RNFL thickness, which remained at 1 month after stoppage in this study, might have been shown to have recovered to the baseline. Furthermore, although GCC analysis is possible with upgraded Cirrus HD-OCT software, the upgraded software was not available at our institute at the time of the study. Changes in GCC thickness might be more dramatic than changes in RNFL thickness, but they might also be less specific, as they involve 3 different innermost retinal layers instead of just one. Future study including foveal GCC or GC-IPL should be conducted with more advanced modalities. Finally, the concurrent effect of isoniazid cannot be ruled out [37].

In conclusion, mean temporal RNFL thickness showed increases after administration of ethambutol. Contrast sensitivity, BCVA and color vision showed no significant change from the baseline. The VFI, VF pattern, and quadrant RNFL thickness could prove useful in assessment of subclinical toxicity. Subclinical ethambutol-induced optic neuropathy was found in a total 22 eyes of 14 patients (i.e., 13% of 168 eyes). Medication duration was shown to be a strong risk factor for occurrence of subclinical toxicity.

## Supporting information

S1 Table Sub-analyses of visual field global indices performed with the data of patients who had visited at a particular time. (DOCX)

S2 Table Sub-analyses of retinal nerve fiber layer thickness performed with the data of patients who had visited at a particular time. (DOCX)
