# Supplementary material for: Longitudinal evaluation of visual function and structure for detection of subclinical Ethambutol-induced optic neuropathy
Source: PLoS One. 2019 Apr 17;14(4):e0215297. doi: 10.1371/journal.pone.0215297 (PMC6469811; doi:10.1371/journal.pone.0215297)
Supplement: S1 Table — (DOCX) [file pone.0215297.s001.docx]

**S1 Table. Sub-analyses of visual field global indices performed with the data of patients who had visited at a particular time**

| **Variable** | **Baseline** | | **Post-administration** | |  |
| --- | --- | --- | --- | --- | --- |
| **MD (dB)** | **Mean** | **SD** | **Mean** | **SD** | **p value*** |
| **1 month (n=139)** | -1.91 | 2.33 | -1.87 | 2.44 | 0.664 |
| **2 months (n=116)** | --2.39 | 2.67 | -1.96 | 2.34 | **0.011** |
| **3 months (n=82)** | -2.61 | 2.95 | -1.41 | 2.40 | **0.000** |
| **4 months (n=70)** | -2.67 | 3.13 | -1.45 | 2.30 | **0.000** |
| **5 months (n=60)** | -2.65 | 2.54 | -1.89 | 2.24 | **0.001** |
| **6 months (n=40)** | -2.18 | 2.36 | -1.02 | 2.05 | **0.000** |
| **7 months (n=16)** | -3.09 | 3.13 | -1.52 | 2.34 | **0.005** |
| **8 months (n=14)** | -3.14 | 2.33 | -1.75 | 3.16 | **0.010** |
| **After stoppage (n=34)** | -2.70 | 2.75 | -1.12 | 1.69 | **0.000** |
| **PSD (dB)** |  |  |  |  |  |
| **1 month (n=139)** | 2.33 | 1.47 | 2.43 | 2.01 | 0.327 |
| **2 months (n=116)** | 2.57 | 1.71 | 2.51 | 1.73 | 0.603 |
| **3 months (n=82)** | 2.74 | 1.91 | 2.28 | 1.39 | **0.009** |
| **4 months (n=70)** | 2.75 | 2.05 | 2.07 | 1.08 | **0.001** |
| **5 months (n=60)** | 2.68 | 1.91 | 2.02 | 0.90 | **0.005** |
| **6 months (n=40)** | 2.15 | 1.20 | 1.70 | 0.60 | **0.023** |
| **7 months (n=16)** | 2.68 | 2.67 | 2.04 | 0.92 | 0.219 |
| **8 months (n=14)** | 2.22 | 0.92 | 1.85 | 0.61 | 0.226 |
| **After stoppage (n=34)** | 3.02 | 2.29 | 2.02 | 1.18 | **0.000** |
| **VFI (%)** |  |  |  |  |  |
| **1 month (n=139)** | 97.14 | 4.09 | 96.83 | 4.37 | 0.441 |
| **2 months (n=116)** | 96.36 | 5.35 | 96.73 | 4.29 | 0.335 |
| **3 months (n=82)** | 95.73 | 6.19 | 97.09 | 4.07 | **0.003** |
| **4 months (n=70)** | 95.83 | 6.57 | 97.53 | 3.34 | **0.004** |
| **5 months (n=60)** | 96.07 | 5.56 | 97.18 | 3.99 | **0.029** |
| **6 months (n=40)** | 96.98 | 5.04 | 98.28 | 3.06 | **0.017** |
| **7 months (n=16)** | 95.94 | 6.95 | 97.75 | 3.61 | 0.081 |
| **8 months (n=14)** | 95.86 | 7.10 | 97.07 | 5.97 | 0.112 |
| **After stoppage (n=34)** | 96.35 | 5.35 | 98.38 | 2.05 | **0.005** |

MD: Mean deviation, PSD: Pattern standard deviation, VFI: Visual field index

n = number

*: Paired-T test
