# Supplementary material for: Longitudinal evaluation of visual function and structure for detection of subclinical Ethambutol-induced optic neuropathy
Source: PLoS One. 2019 Apr 17;14(4):e0215297. doi: 10.1371/journal.pone.0215297 (PMC6469811; doi:10.1371/journal.pone.0215297)
Supplement: S2 Table — (DOCX) [file pone.0215297.s002.docx]

**S2 Table. Sub-analyses of retinal nerve fiber layer thickness performed with the data of patients who had visited at a particular time**

| **Variable** | **Baseline** | | **Post-administration** | |  |
| --- | --- | --- | --- | --- | --- |
| **Average** | **Mean** | **SD** | **Mean** | **SD** | **p value*** |
| **1 month (n=152)** | 101.00 | 14.49 | 101.93 | 12.63 | 0.313 |
| **2 months (n=124)** | 101.11 | 12.86 | 101.51 | 13.43 | 0.708 |
| **3 months (n=88)** | 98.95 | 16.26 | 99.89 | 13.41 | 0.429 |
| **4 months (n=76)** | 100.66 | 14.07 | 100.11 | 13.19 | 0.502 |
| **5 months (n=60)** | 99.36 | 12.84 | 99.02 | 11.02 | 0.733 |
| **6 months (n=40)** | 104.88 | 15.18 | 105.65 | 15.38 | 0.683 |
| **7 months (n=16)** | 96.81 | 11.93 | 96.19 | 11.49 | 0.464 |
| **8 months (n=14)** | 97.92 | 10.24 | 97.00 | 11.37 | 0.303 |
| **After stoppage (n=42)** | 97.25 | 10.47 | 97.80 | 10.86 | 0.569 |
| **Superior** |  |  |  |  |  |
| **1 month (n=152)** | 125.95 | 16.56 | 127.01 | 19.95 | 0.330 |
| **2 months (n=124)** | 125.43 | 18.15 | 126.14 | 19.20 | 0.572 |
| **3 months (n=88)** | 123.83 | 18.31 | 123.54 | 20.12 | 0.863 |
| **4 months (n=76)** | 124.84 | 19.64 | 122.87 | 19.45 | 0.152 |
| **5 months (n=60)** | 123.69 | 16.79 | 122.96 | 16.81 | 0.603 |
| **6 months (n=40)** | 131.59 | 18.98 | 131.34 | 20.38 | 0.923 |
| **7 months (n=16)** | 121.19 | 18.52 | 118.56 | 16.93 | 0.157 |
| **8 months (n=14)** | 122.33 | 10.21 | 120.17 | 10.87 | 0.139 |
| **After stoppage (n=42)** | 119.95 | 15.75 | 119.63 | 17.13 | 0.827 |
| **Inferior** |  |  |  |  |  |
| **1 month (n=152)** | 128.69 | 22.32 | 129.38 | 22.91 | 0.694 |
| **2 months (n=124)** | 128.14 | 23.30 | 130.01 | 22.00 | 0.172 |
| **3 months (n=88)** | 125.68 | 23.47 | 126.38 | 21.95 | 0.804 |
| **4 months (n=76)** | 126.79 | 20.52 | 127.34 | 20.97 | 0.581 |
| **5 months (n=60)** | 122.68 | 23.50 | 123.54 | 19.59 | 0.705 |
| **6 months (n=40)** | 131.68 | 28.65 | 135.53 | 23.12 | 0.291 |
| **7 months (n=16)** | 125.44 | 17.64 | 126.75 | 19.85 | 0.544 |
| **8 months (n=14)** | 129.75 | 14.85 | 127.83 | 15.71 | 0.110 |
| **After stoppage (n=42)** | 120.33 | 24.84 | 123.18 | 20.22 | 0.319 |
| **Temporal** |  |  |  |  |  |
| **1 month (n=152)** | 78.91 | 17.42 | 78.41 | 16.62 | 0.530 |
| **2 months (n=124)** | 78.16 | 17.51 | 78.61 | 16.06 | 0.538 |
| **3 months (n=88)** | 78.16 | 19.31 | 80.32 | 18.68 | 0.060 |
| **4 months (n=76)** | 78.06 | 17.96 | 78.34 | 20.97 | 0.794 |
| **5 months (n=60)** | 76.54 | 17.49 | 78.71 | 17.22 | 0.102 |
| **6 months (n=40)** | 78.71 | 20.36 | 82.76 | 19.43 | **0.014** |
| **7 months (n=16)** | 74.25 | 16.70 | 75.00 | 15.54 | 0.840 |
| **8 months (n=14)** | 70.08 | 13.24 | 71.17 | 16.95 | 0.699 |
| **After stoppage (n=42)** | 76.00 | 14.13 | 78.68 | 15.22 | 0.116 |
| **Nasal** |  |  |  |  |  |
| **1 month (n=152)** | 72.80 | 15.89 | 72.72 | 15.50 | 0.812 |
| **2 months (n=124)** | 71.64 | 15.03 | 71.92 | 13.71 | 0.933 |
| **3 months (n=88)** | 71.11 | 15.92 | 70.02 | 14.20 | 0.428 |
| **4 months (n=76)** | 73.21 | 16.85 | 71.97 | 13.50 | 0.354 |
| **5 months (n=60)** | 72.55 | 16.47 | 70.75 | 15.04 | 0.274 |
| **6 months (n=40)** | 74.38 | 19.15 | 74.29 | 16.69 | 0.975 |
| **7 months (n=16)** | 65.88 | 13.68 | 67.19 | 16.99 | 0.540 |
| **8 months (n=14)** | 71.08 | 16.13 | 70.00 | 16.25 | 0.557 |
| **After stoppage (n=42)** | 68.55 | 13.66 | 69.28 | 17.12 | 0.640 |

n = number

*: Paired-T test
